# Cultural Adaptation of an Evidence-Informed Psychosocial Intervention to Address the Needs of PHIV+ Youth in Thailand

**DOI:** 10.1007/s40609-017-0100-x

**Published:** 2017-07-24

**Authors:** Gisselle Pardo, Chutima Saisaengjan, Priya Gopalan, Jintanat Ananworanich, Sudrak Lakhonpon, Danielle Friedman Nestadt, Torsak Bunupuradah, Claude Ann Mellins, Mary McKernan McKay

**Affiliations:** 1 McSilver Institute for Poverty Policy and Research, New York University Silver School of Social Work, 41 East 11th Street, 7th Floor, New York, NY 10003 USA; 2 The Children and Youth Program, SEARCH, HIV-NAT, The Thai Red Cross AIDS Research Centre, Bangkok, Thailand; 3 Present Address: U.S. Military HIV Research Program, Henry M. Jackson Foundation for the Advancement of Military Medicine, Bethesda, USA; 4 HIV Center for Clinical and Behavioral Studies, New York State Psychiatric Institute and Columbia University, New York, USA; 5 The HIV Netherlands Australia Thailand Research Collaboration (HIV-NAT), The Thai Red Cross AIDS Research Centre, Bangkok, Thailand

**Keywords:** Cultural adaption, Curriculum development, Family process variables, Medication adherence, HIV risk behaviors, Youth behavioral health outcomes

## Abstract

Globally, pediatric HIV has largely become an adolescent epidemic. Thailand has the highest HIV prevalence in Asia (1.2%), with more than 14,000 children living with HIV. There is growing demand for evidence-based psychosocial interventions for this population that include health and mental health support and sexual risk reduction, which can be integrated into HIV care systems. To address this need, a multidisciplinary team of Thai and US researchers adapted an existing evidence-informed, family-based intervention, The Collaborative HIV Prevention and Adolescent Mental Health Program + (CHAMP+), which has been tested in multiple global trials. Using community-based participatory research methods, changes to the intervention curriculum were made to address language, culture, and Thai family life. Involvement of families, youth, and stakeholders in the adaptation process allowed for identification of salient issues and of program delivery methods that would increase engagement. Participants endorsed using a cartoon-based curriculum format for fostering discussion (as in CHAMP+ South Africa) given stigma around discussing HIV in the Thai context. The Thai version of CHAMP+ retained much of the curriculum content incorporating culturally appropriate metaphors and story line. Sessions focus on family communication, coping, disclosure, stigma, social support, and HIV education. This paper explores lessons learned through the adaption process of CHAMP+ Thailand that are applicable to other interventions and settings. It discusses how culturally informed adaptations can be made to interventions while maintaining core program components.

## Introduction

Globally, pediatric HIV has largely become an adolescent and young adult epidemic. Improvements in antiretroviral treatment (ART) and increased resource allocation have reduced perinatal transmission of the virus and enabled millions of perinatally HIV-infected (PHIV+) children, once not expected to live more than a few years, to lead full, healthy, and productive lives, albeit with a chronic, highly stigmatized condition. In 2013, an estimated 3.3 million children were living with PHIV worldwide, nearly two thirds of whom were adolescents between the ages of 10 and 19 years (Joint United Nations 2013). The vast majority of PHIV+ children live in low resource settings, where efforts to prevent PHIV were seriously delayed and treatment services often focus exclusively on ensuring access to medications. Scarce attention is paid to psychosocial and other ancillary needs of this vulnerable population. With larger numbers of children surviving into their teen years, there are critically important needs to be met in areas of adherence, psychosocial support, and sexual health (Mofenson and Cotton 2013).

Global studies have revealed high rates of mental health challenges, as well as sexual and substance use risk behavior, among PHIV+ teens (Malee et al. 2011; Mellins and Malee 2013; Elkington et al. 2010). Neglect of the psychosocial and behavioral health needs of PHIV+ children may contribute to non-adherence to medication in adolescence, leading to higher viral loads and multi-drug-resistant mutations of the virus (Bucek et al. 2016; Cruz and Cardoso 2015; Mellins et al. 2011; Naar-King et al. 2006). These are both individual and public health concerns. There is a considerable risk of further transmission of virus mutations via sexual contact, since studies in the USA reveal that two thirds of PHIV+ teens engaged in unprotected sex, many with unsuppressed viral loads (Tassiopoulos et al. 2013). Moreover, there was a 50% increase in AIDS-related deaths among adolescents between 2005 and 2012, even though mortality rates related to HIV dropped for all other age groups (Joint United Nations 2013). It has become increasingly clear that medications and biomedical interventions alone will not contain the epidemic. Psychosocial interventions are essential to HIV treatment, particularly among adolescents (Kennedy et al. 2010).

### HIV in Thailand

Although the global AIDS epidemic has been concentrated in Africa, a significant number of HIV-infected children live in Asia. Thailand has the highest HIV prevalence in Asia (1.2%) (World Bank 2015), with more than 14,000 children living with HIV (UNICEF 2014). Although Thailand’s aggressive response to HIV includes provision of free and timely medication, there is growing concern that HIV-related stigma and discrimination is unaddressed, resulting in testing and treatment gaps (UNICEF 2014). Studies of HIV+ youth in Thailand, including PHIV+ and behaviorally infected youth, indicate that some youth are engaging in sexual risk behaviors, with low levels of condom use and high rates of mental health problems (Lolekha et al. 2015; Rongkavilit et al. 2007; Rongkavilit et al. 2010). There is a growing demand for psychosocial interventions that can be integrated into HIV care systems across the world, particularly in countries like Thailand, where knowledge and use of evidence-based programs that include mental health support for HIV+ youth remain limited (Jenkins and Kim 2004). To address the unmet need for these interventions in Thailand, a group of US and Thai researchers and care providers set out to adapt an existing evidence-informed intervention to be used in the Thai context. This paper describes the adaptation process of The Collaborative HIV Prevention and Adolescent Mental Health Program (CHAMP+) to the Thai context.

### Background of the CHAMP and CHAMP+ Model

The Collaborative HIV Prevention and Adolescent Mental Health Program (CHAMP) is an evidence-informed, family-based intervention, originally developed for HIV-negative early adolescents in Chicago and targets prevention of risk behaviors that might lead to HIV-infection (McKay and Paikoff 2007; Petersen et al. 2010; McKay et al. 2007; Mellins et al. 2014). The goal was to reach young people before they were engaged in risk-taking behaviors, as prevention is much more effective than efforts to reduce behaviors already in place (McKay and Paikoff 2007). CHAMP is designed to be delivered via multiple family groups of youth and their adult caregivers, in 10 to 12 meetings, where topics such as communication, parenting, HIV knowledge, puberty, and social support are discussed in separate, as well as combined, groups of caregivers and adolescents. CHAMP has been tested in multiple trials across the globe, with positive effects on family process variables (e.g., communication, parent involvement, and support), as well as youth behavioral health outcomes (e.g., improved self-esteem and reduced participation in risk behaviors) (McKay and Paikoff 2007; McKay et al. 2007; Bell et al. 2008; Bhana et al. 2010; McKay et al. 2004), and is now recognized as an effective tool to promote mental health and reduce sexual risk behaviors for early adolescents affected by HIV (Substance Abuse and Mental Health 2012).

Given the many unmet needs of PHIV+ early adolescents and their caregivers in the USA and the positive outcomes found in CHAMP studies, the CHAMP model was adapted to address issues around adherence, mental health, and sexual risk behavior for PHIV+ youth in New York (McKay et al. 2007). Many of the topics from the original CHAMP were retained. However, CHAMP+ also included content specific to growing up and living with HIV, such as medication adherence, feelings about HIV and identity, disclosure, and stigma. CHAMP+ has been adapted and tested in New York, South Africa, and Argentina in multiple randomized control trials, which have shown positive effects on family process variables, youth behavioral health outcomes and improved adherence among PHIV+ youth (McKay et al. 2007; Bhana et al. 2010).

Using community-based participatory research (CBPR) methods, the CHAMP model has been adapted across a range of different cultural context. In South Africa, adaptations were needed to facilitate discussion of sensitive topics given considerable stigma around discussions of HIV and sexual behavior in families. A collaborative team in South Africa designed a cartoon-based curriculum for the CHAMP and CHAMP+ programs. The cartoon creates distance that encourages discussion of culturally sensitive issues among the story characters prior to participants talking about themselves. The cartoons also increase accessibility for participants who may have limited literacy skills.

### CHAMP+ Thailand

Based on the positive findings in other global studies of CHAMP+ and the needs of PHIV+ youth in Thailand, a team of providers and researchers in Thailand sought to adapt and test the intervention in their country, where few evidence-based programs for this population exist. However, concerns about cultural differences between Thailand and places where CHAMP+ had been previously implemented highlighted the need for extensive formative and adaptive work before implementation.

In 2013, with support from Therapeutics Research Education and AIDS Training in Asia (Treat Asia), researchers in Bangkok conducted formative research to explore and analyze the local needs of PHIV+ youth and their families in order to determine whether the CHAMP model could be adapted and used in their setting. The first phase focused on individual interviews and focus groups to identify salient stressors and needs of PHIV+ youth in Thailand. Phase 2 used this data and CBPR processes to develop a culturally sensitive CHAMP+ intervention curriculum and facilitator’s manual ready for testing through a pilot trial implementation. This paper describes the phase 2 process of adapting the CHAMP+ intervention in Thailand.

## Methods

The process of adapting the CHAMP+ intervention occurred in two phases using qualitative methods. Focus group methods were selected to capture the collective perceptions of a community and would allow for exploration of the social, cultural, and environmental conditions that impact coping and perceptions about HIV. Key informant in-depth interviews were used to supplement focus group data with both sources used to guide planning and implementation of the adapted program.

The study took place at the Thai Red Cross AIDS Research Centre, a publicly funded clinic serving families affected by HIV in Bangkok, Thailand. All the necessary IRB permissions were obtained from the respective institutions, Thai Red Cross and New York University.

Purposive sampling was used. The inclusion criteria for adolescents was (1) being between the ages of 12 and 16 years, (2) having a diagnosis of perinatally acquired HIV, (3) receiving care are at the Thai Red Cross, and (4) being aware of their HIV diagnosis. The caregiver inclusion criteria was (1) adult over 18 years old (2) living with and responsible for caring for the adolescent and (3) aware of the adolescent’s HIV status. Participants who met the inclusion criteria were invited to participate in the study by Thai research staff during medical visits or via phone calls. A total of 12 families provided informed consent to participate.

### Phase 1

In order to understand the unique risk and protective factors for Thai PHIV+ youth, Thai family dynamics, Thai family daily life, and the impact of HIV on Thai families, the adaption process began with formative research. This formative research phase consisted of focus groups with Thai PHIV+ youth and their caregivers. One focus group for youth was conducted with a total of 10 participants and one focus group for caregivers with a total of eight participants. Groups were conducted in Thai by staff known to participants. Researchers explored key themes around growing up with HIV, family communication, the role of religion and culture in families affected by HIV, HIV knowledge, adherence, and stigma. In addition to the focus groups, six key informant interviews were conducted with clinic staff and other stakeholders. Based on results from phase 1, the Thai and US research staff began to make adaptations to the intervention curriculum. The methods and results of phase 1 are described in greater detail in a paper by Nestadt et al. (2017) currently in submission.

### Phase 2 Curriculum Development

Integrating the results from phase 1, two Thai researchers and an artist, who is the parent of a PHIV+ youth, outlined the topics for each session, then wrote and drew the first draft of the Thai intervention cartoon. This draft was written in Thai titled “Tang-Sai-Mai” (“The New Road”) and consisted of 10 sessions.

The PHIV+ youth and caregivers who consented to participate in the study were then invited back for an additional focus group to review this first draft of the intervention curriculum. A total of ten adolescents, ten caregivers, and the cartoon artist participated. The group was facilitated in Thai by two Thai staff and two Thai researchers known to participants. Researchers used a semi-structured interview guide and provided copies of the draft intervention. This focus group review of the curriculum draft lasted half a day. It took place on a weekend to accommodate work and school schedules. The focus group went through the curriculum draft session by session; most sessions were reviewed in separate adolescent and caregiver groups, with the final session reviewed as a large group. The adolescents who participated ranged in age from 13 to 16 years and included five males and five females. The caregivers included two biological fathers, four biological mothers, two adoptive mothers, and two grandmothers. There were two caregivers in phase 2 who did not participate in the phase 1 focus group.

Researchers explored participant’s thoughts on the use of a cartoon format and multiple family group model as well as what difficulties may come up in situating this type of intervention within the Thai context. Participants provided feedback on the cartoon storyline, including the development of the main characters, session discussion questions, games and activities, management of topics within each session and order of topics, as well as length and timing of intervention delivery. Researchers explored how relatable the character and the challenges he faced were to participant’s own lives.

Focus groups were audio recorded, then participants’ comments summarized in Thai and translated to English by the Thai research team. Based on the information and feedback from the phase 2 focus groups, the Thai- and US-based research team collaboratively revised and finalized the intervention curriculum.

## Results

Analysis of the focus group and individual key informant interviews of phase one revealed psychosocial challenges Thai youth and families living with HIV face, particularly related to adherence, disclosure, stigma, and parent-child communication, as well as some approaches used to deal with them. Some were similar to findings from other settings, while others were specific to the Thai context. Overall, focus groups and interviews indicated that families need additional support and education. Participants also indicated that a cartoon format, similar to that used in South Africa, would be most appropriate for the Thai context given significant stigma and reluctance to discuss HIV, sexuality, and family secrets.

Integration of participant feedback from phase 2 led to changes in the storyline and reordering of session topics. For example, loss and bereavement was moved to a later session since participants expressed needing time to build trust before addressing difficult topics. Participants felt it was important that the curriculum focus on other aspects of youth identity beyond their HIV diagnosis as well as provide hope for the future; therefore, a session on identity and empowerment was added. The intervention was also designed to be delivered one weekend day per month over 6 months, and, with the exception of the final session, two sessions would be covered each day. The results of phase 2 informed the development of a structured facilitator’s manual to accompany the cartoons. For each session, the manual guides facilitators through the cartoon storyline for that session, related activities, discussion questions, probes, and tips for engaging participants. The title was also changed to “Walking Together.” The final draft of the intervention curriculum consists of 11 sessions discussed in detail below.

### Session Content and Description

Walking Together focuses on the story of Tam, a 12-year-old HIV+ boy, and his mother, Joom, who is also HIV+. His father died several years earlier. At the start of the story, Tam has just learned he has HIV. No one in their neighborhood, at Joom’s workplace, or at Tam’s school knows that he and his mother have HIV. The creation of the characters and the family configuration was based on input from Thai PHIV+ youth and families. At present in Thailand, it is not uncommon for a PHIV+ youth to be living with their biological parent, unlike in other CHAMP+ settings where caregivers of PHIV+ youth are often extended family.

#### Sessions 1 and 2

**Fig. 1:**
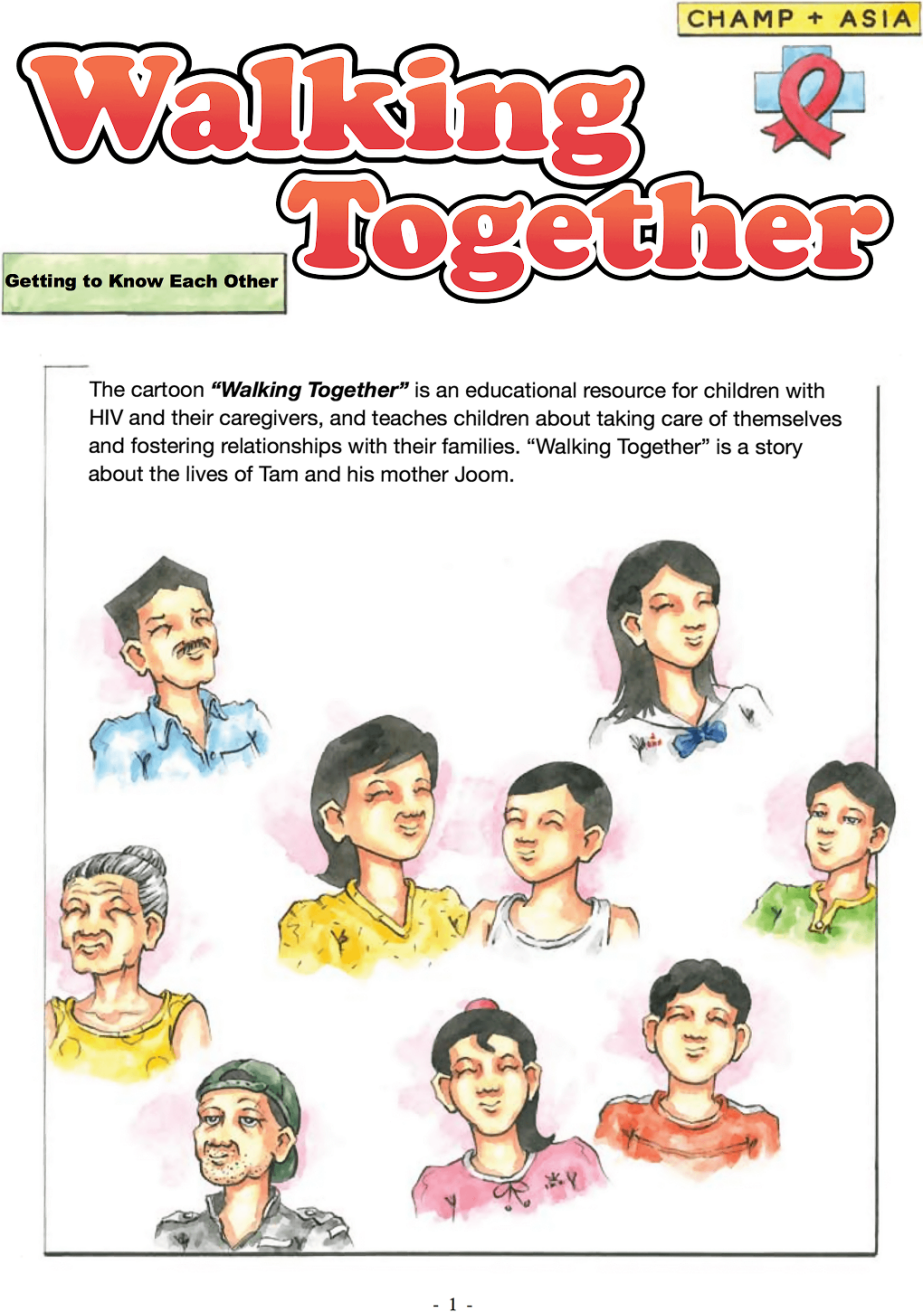
Walking Together: Getting to Know Each Other cover

**Fig. 2:**
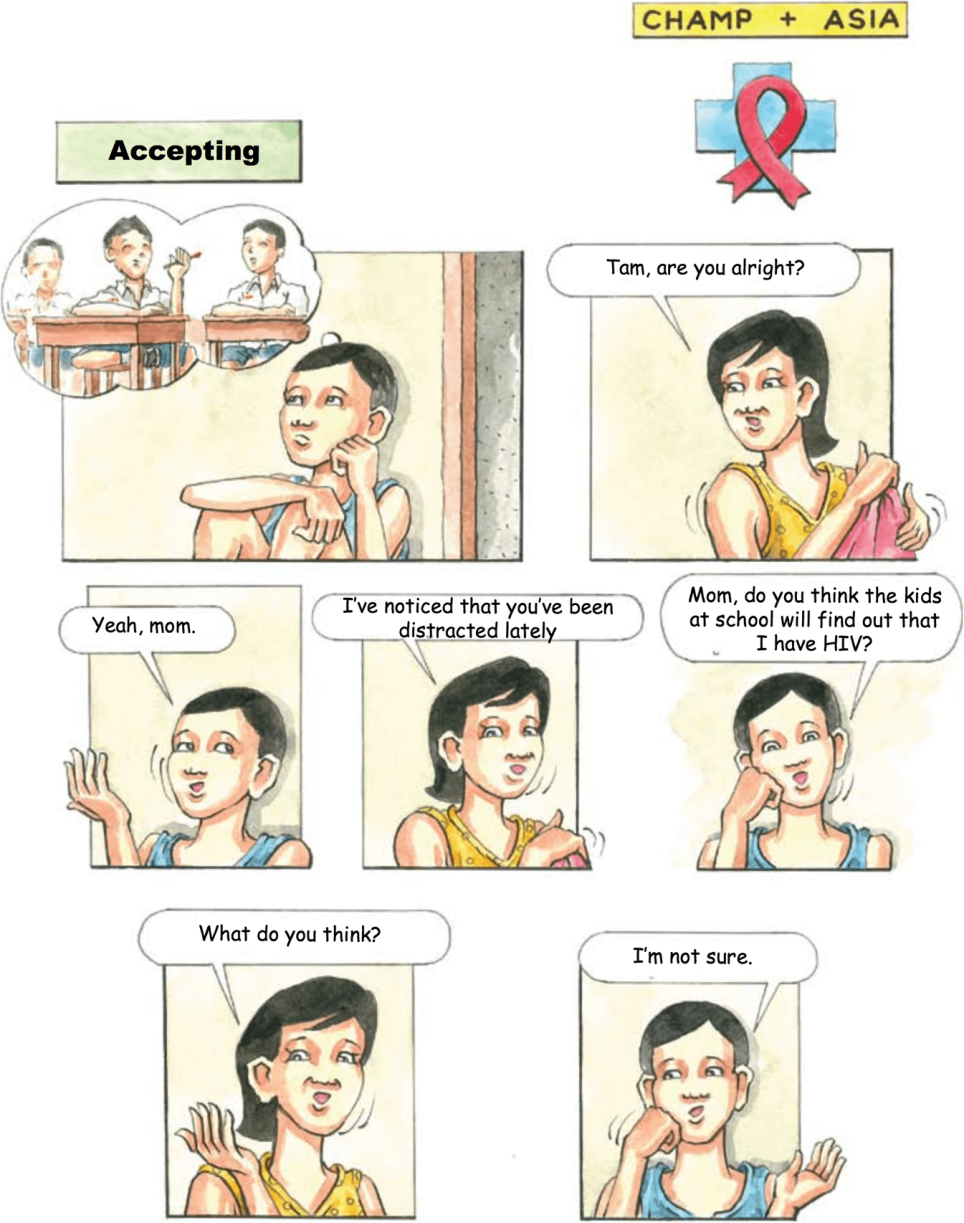
“Accepting”

The first two sessions of Walking Together, which are called “Getting to Know Each Other” (Fig. 1) and “Accepting,” (Fig. 2) are conducted on the first day of the program. They introduce participants to the program format, the other participants, and the facilitators and establish rules for group interaction. Establishing rapport and creating a safe environment to enable discussion is an important step in the multiple family group. The second session introduces families to Tam and Joom and then uses discussion questions and icebreaker activities to encourage participants to share their concerns about growing up with HIV.

The Thai adaptation begins with a more gradual initiation to the group process than previous versions of CHAMP+ to accommodate the cultural barriers in Thailand toward disclosure and sharing. As most of the families who participated in initial focus groups had never publicly acknowledged their concerns about living with HIV, the cartoon and activities aim to help families feel more at ease discussing HIV and their feelings.

#### Sessions 3 and 4

The third and fourth sessions focus on the importance of communication and the stigma around HIV. Session three, “Opening Up,” starts with Joom asking Tam to do some chores, which he does not do, resulting in yelling between the two and later an attempt to resolve their conflict. Results of phase 1 focus groups indicated that in Thai culture, there is a great deal of hierarchy and parents must be respected and obeyed. At the same time, families in the focus groups expressed a strong desire to improve communication between parent and child. This session highlights strategies to improve communication between parents and children within the context of Thai culture.

**Fig. 3:**
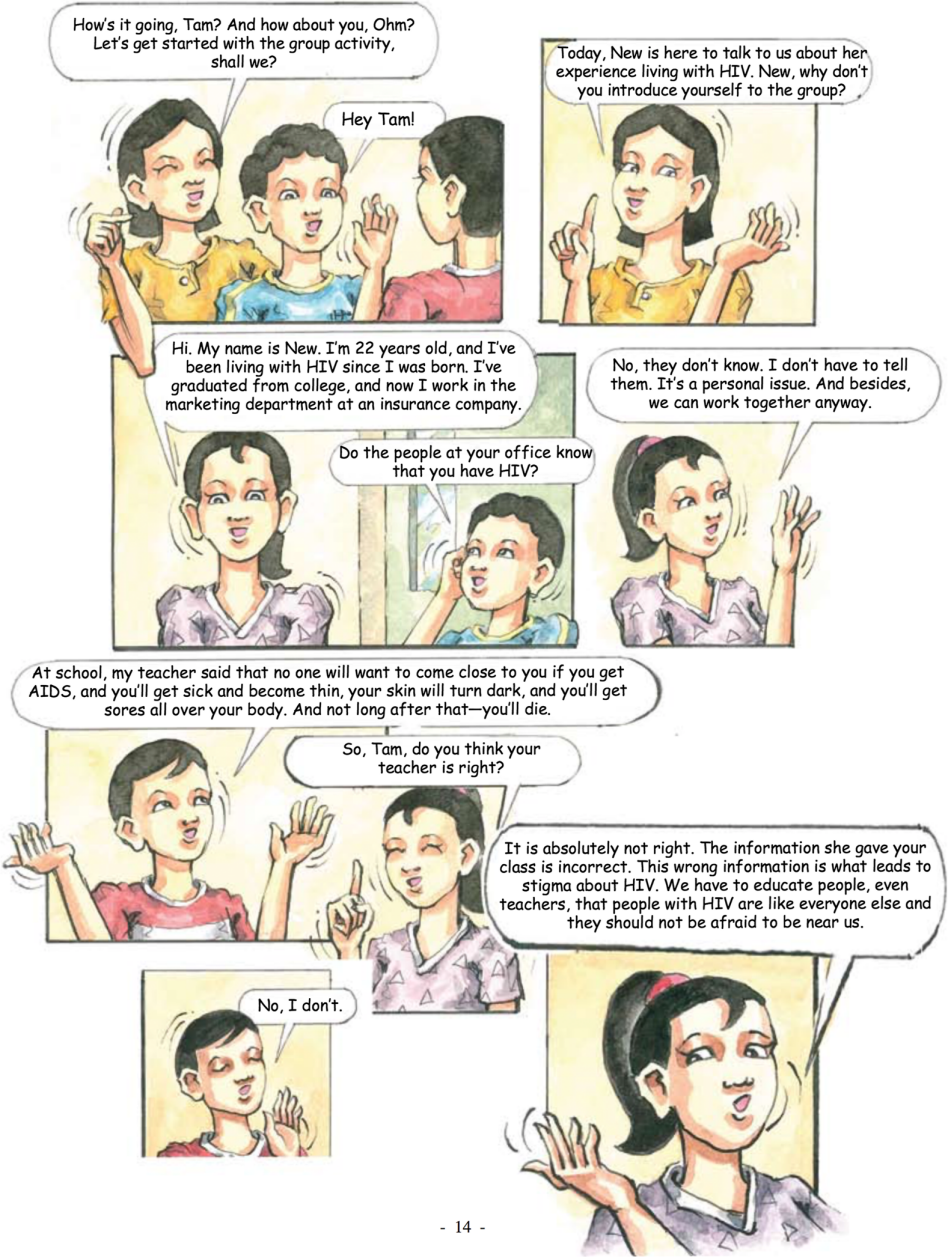
“Understanding Each Other”

Session four, “Understanding Each Other” (Fig. 3), focuses on coping with stigma and discrimination. In this episode, a teacher and students at Tam’s school make incorrect and negative statements about people with HIV, which upsets Tam. He talks to his mom and his clinic peer support group, who share similar experiences with HIV stigma and discrimination. Phase 1 focus group participants reported widespread misunderstanding about HIV in Thai society, leading to shame and discrimination. Thus, this session was designed to give participants a space to discuss and identify ways to cope with stigma and fears of rejection.

#### Sessions 5 and 6

**Fig. 4:**
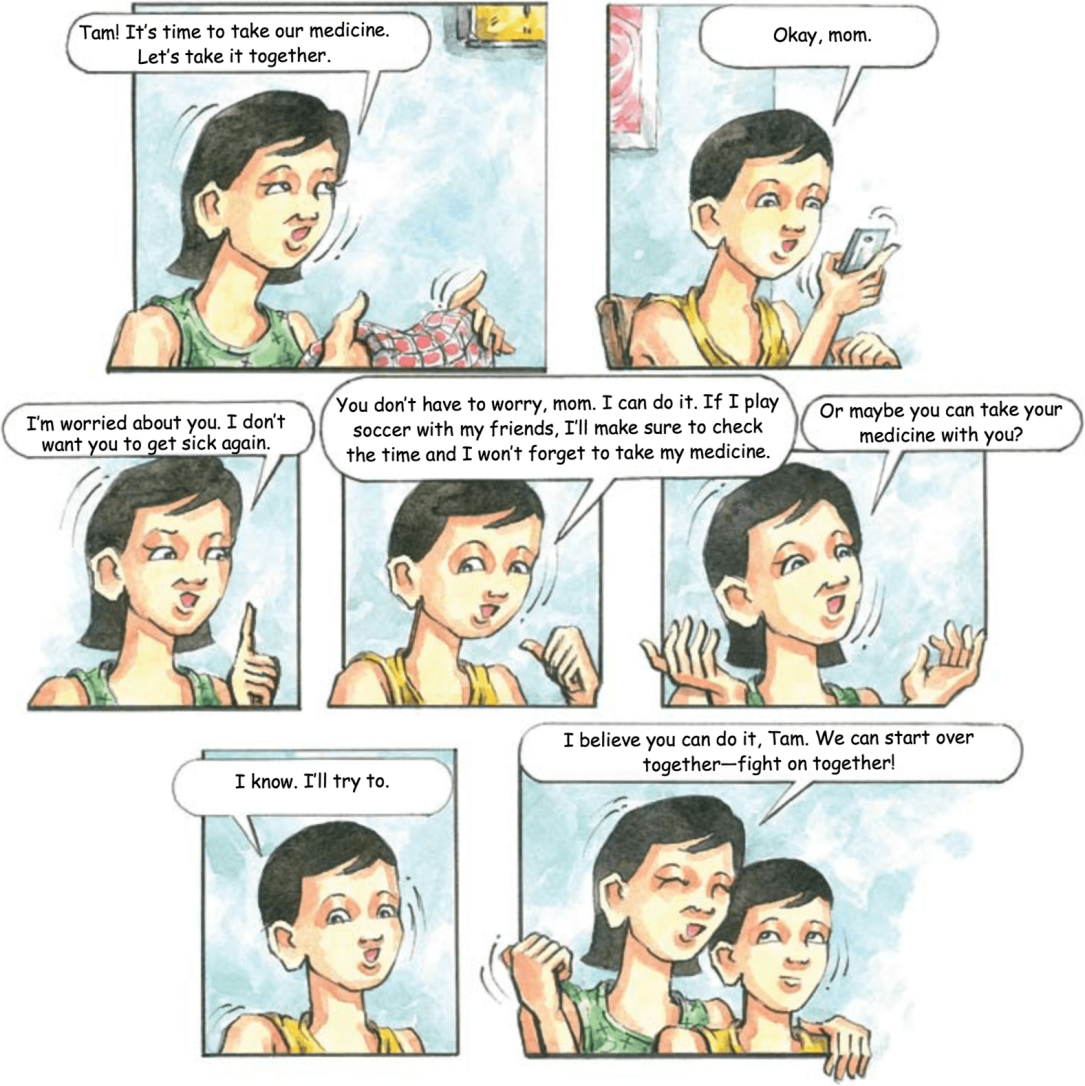
“HIV Knowledge and Treatment”

The fifth session, “Working Together: HIV Knowledge and Treatment” (Fig. 4), aims to reinforce understanding of HIV treatment and the importance of adherence to treatment. In this session, Tam forgets to take his medicine, and his viral load increases. Focus group youth participants had identified parental support as being important in maintaining adherence. In this session, Tam’s mother suggests they take their medication together, demonstrating one way parents can support their children’s adherence. The session’s discussion questions focus on strategies to address barriers to adherence.

This session is followed by “Holding Hands: HIV Disclosure,” which focuses on improving comfort and preparation for disclosure of participants’ HIV status to others. Since families in phase 1 focus groups identified knowing how to disclose to a boyfriend/girlfriend or partner as a major concern, this session’s cartoon story focuses on mom, Joom, disclosing her status to a new partner, named Yo. Through a disclosure role-playing activity, facilitators help families voice and share concerns about disclosure, past experiences, and how to approach future disclosure.

One common suggestion from families on how to disclose is to give your partner a hypothetical situation first to see how they react before telling them about your own status. This idea was incorporated into the cartoon storyline where mom Joom uses a TV show to facilitate general discussion about HIV with her partner before disclosing her own status.

#### Sessions 7 and 8

Session seven, “Walking Together: Loss and Bereavement,” deals with how to cope with grief and loss. In this session, Tam and his mother discuss their feelings of loss after Tam’s father died, as verbalizing feelings of loss is not customary in Thai culture. This session focuses on the healing effects of exploring and sharing emotions and providing mutual support. In Thai culture, it is important to let loved ones, who have died, know that you remember them and are doing well. In this session, Tam has a watch that belonged to his father as a way to remember him and focuses on positive memories of his father.

In session eight, “Being Triumphant: Dealing with Risk Behavior and Peer Pressure,” Tam is pressured by friends to attend a party and smoke a cigarette. Caregivers are asked to reflect on their own experiences in adolescence, and facilitators offer tools and strategies for helping children navigate risky situations. Children discuss risky situations and how to handle peer pressure.

#### Sessions 9 and 10

In session nine, “Finding Love: Dealing with Puberty and Sexual Relationships,” Tam asks a girl at school to be his girlfriend. Tam’s mother is upset because she does not feel that Tam should be dating. Focus group caregiver respondents expressed concerns about their PHIV+ youth starting to date. Therefore, this session’s activities and discussion focus on adolescent development and how parents and youth can discuss the sensitive topic of dating.

Session ten, “Positive Lives: Identity and Empowerment,” focuses on identity, goals, and hopes for the future. In this session, Tam explores career and vocational plans. Discussion and group activities focus on helping youth look toward the future and explore their identity, beyond being HIV positive. In this session, the goal is to create a sense of hopefulness and empowerment for children and caregivers. Building future orientation and identifying future vocations for PHIV+ youth was critically important to the Thai families and researchers involved in developing the program.

#### Session 11

In the eleventh and final session, “Reaching Your Dream: Peer Group Support,” Tam; Joom; and her partner, Yo attend a family activity at the hospital, where families share their hopes for the future and how important having a support network has been. Participants are asked to reflect on their own social supports and their experience in CHAMP+. Facilitators use the session to bring the program to a close and express appreciation to participants. Since Thai culture values the expression of gratitude and gift giving, this session incorporates an activity where everyone receives a small token of appreciation from another person in order to create a lasting bond.

## Discussion

In the global fight against HIV and AIDS, there is a critical need for evidence-informed, developmentally timed interventions that prevent risk behavior and promote mental health among PHIV+ youth as they transition from childhood to adolescence and eventually young adulthood. CHAMP+ is one of the few evidence-informed, family-based interventions designed to prevent risk behavior and build both individual and family-level protective factors. In order to ensure cultural competency and congruence, a process of adapting the original intervention was undertaken in collaboration with stakeholders before implementing CHAMP+ in a new global setting. Adapting interventions in partnership with communities also enhances the community’s commitment and the chances that the program will be sustained overtime (Castro et al. 2004). The use of community-based participatory research (CBPR) methods has been key to adapting CHAMP+ across diverse global settings (Mellins et al. 2014; McKay et al. 2007).

The process described in this paper allowed researchers and stakeholders to adapt the intervention to the Thai context, to adjust the “fit,” without compromising the integrity of the intervention. The involvement of families, youth, and other stakeholders in the adaptation process allowed for the identification of salient issues for Thai families and of program delivery methods that would increase receptiveness and engagement.

Despite cultural and contextual differences between the various communities where CHAMP+ has been adapted, the core elements of the program (e.g., multiple family group format, 10–12 sessions, separate and combined caregiver and youth groups) were retained. In addition, there have been common universal topics identified by stakeholders in New York, Argentina, South Africa, and Thailand that are present across all versions of the program curriculum (e.g., stigma, disclosure, adherence, family communication). The adaption process ensures these topics are addressed using culturally acceptable teaching methods and in the native language of each community. For example, the use of cartoons in South Africa facilitated the introduction of sensitive topics and used the cultural tradition of “storytelling.” The cartoon format appealed to Thai families as it allowed discussion of culturally taboo topics through a story and allowed them to talk about the characters, instead of themselves, before they felt ready to share.

Although there are universal themes affecting families in each setting where CHAMP+ has been implemented, changes were needed to program content, process, and delivery as noted above. For example, when addressing HIV knowledge in South Africa, greater emphasis was placed on using local metaphors to convey messages about HIV (such as talking about the body as a castle under attack by the virus). In Thailand, knowledge about HIV was introduced around undoing misinformation about HIV that is prevalent in Thai culture, including transmission routes and that HIV is punishment for being a bad person. The South African intervention addresses disclosure from the perspective of the child struggling to tell a peer, while the Thai focuses more on parental experience of disclosure to a partner. The topic of loss is addressed in every adaption of CHAMP+. In South Africa, the session on loss occurs first, addressing it early before moving on to the other topics. In Thailand, the session on loss is much later, because stakeholders felt participants would be more willing to engage with the topic once they were more familiar with the program and with each other. It was also important to Thai stakeholders that each session ends on a positive note. Therefore, CHAMP+ Thailand strategically uses games at the beginning, middle, and end of the day’s program to facilitate bonding and lighten the mood of participants after addressing difficult topics.

There are several limitations to this study that must be noted. Participants were only recruited from one site located in a major urban area, and therefore, findings may not be representative of families living in more rural areas. In phase 2, only one focus group was conducted with 21 participants to review and revise the intervention curriculum. Given this small sample size, we cannot generalize to the larger Thai PHIV+ youth population. Data from this focus group was summarized and translated to English by Thai research team; therefore, some meaning may have been lost in translation. Despite the limitations, this study adds knowledge to the psychosocial needs of PHIV+ Thai youth and their families. This paper also represents one of the few to describe the process of adaptation for a specific intervention program, which is important for those engaged in developing interventions that can be tailored to different contexts.

As a next step, the feasibility and effectiveness of the adapted cartoon-based Thai CHAMP+ will be tested through a small-scale RTC pilot in Thailand. Those outcomes will inform future refinement and adaption of the CHAMP+ program in Thailand.

## Conclusion

In summary, there are several lessons learned through the adaptation process of CHAMP+ across settings that are applicable to other interventions and settings.

- The adaption process must start with a needs assessment to understand the local culture and context and the unique risk and protective factors of the population.
- True collaboration and communication between all the partners (US consultants, Thai families, researchers, and clinical providers) is an essential part of the process.
- Adaptation of the curriculum must include culturally acceptable delivery methods to make it relevant and acceptable to the local context.
- The delivery format should be adapted to address the real world needs of families.
